# SARS-CoV-2 Variant-Specific Antibodies in Vaccinated Inflammatory Bowel Disease Patients

**DOI:** 10.3390/vaccines13060595

**Published:** 2025-05-30

**Authors:** Eva Ulla Lorentzen, Richard Vollenberg, Rieke Neddermeyer, Michael Schoefbaenker, Eike R. Hrincius, Stephan Ludwig, Phil-Robin Tepasse, Joachim Ewald Kuehn

**Affiliations:** 1 Institute of Virology, University of Muenster, Von-Stauffenberg-Str. 36, D-48151 Muenster, Germany; r_nedd01@uni-muenster.de (R.N.); m_scho79@uni-muenster.de (M.S.); hrincius@uni-muenster.de (E.R.H.); ludwigs@uni-muenster.de (S.L.); kuehnj@uni-muenster.de (J.E.K.); 2 Department of Medicine B for Gastroenterology, Hepatology, Endocrinology and Clinical Infectiology, University Hospital Muenster, D-48149 Muenster, Germany; richard.vollenberg@ukmuenster.de (R.V.); phil-robin.tepasse@ukmuenster.de (P.-R.T.)

**Keywords:** SARS-CoV-2, IBD patients, anti-TNF therapy, vaccination, humoral immune response, variant-specific antibodies

## Abstract

**Background/Objectives:** Patients suffering from inflammatory bowel diseases (IBDs) undergoing treatment with anti-TNF antibodies mount a diminished humoral immune response to vaccination against SARS-CoV-2 compared to healthy controls. The characterization of variant-specific immune responses is particularly warranted among immunosuppressed patients, where reduced responses may necessitate further medical interventions. **Methods:** This pilot study investigated the humoral immune response of vaccinated IBD patients on anti-TNF medication and a comparable group of healthy individuals against the viral variants Alpha, Beta, Gamma, Delta, and Omicron BA.1 and BA.5. While total IgG antibodies targeting the receptor binding site of the spike protein of SARS-CoV-2 were quantified using a chemiluminescence microparticle immunoassay (CMIA), their potential neutralizing capacity was determined using commercial and variant-specific in-house surrogate virus neutralization tests (sVNTs) against a variant-specific in-house VSV-pseudotyped virus neutralization test (pVNT) as the gold standard. **Results:** Employing variant-specific assays recapitulated the immune escape functions of virus variants. Conspicuously, antibody reactivity against Alpha and Omicron BA.1 and BA.5 was strikingly poor in IBD patient sera post-initial vaccination compared to healthy individuals. A comparison of the diagnostic performance of assays with the pVNT revealed that identification of patients with inadequate humoral responses by CMIA and sVNT may require adjustments to cut-off values and end-point titration of sera. Following adaptation of cut-off values, patient sera exhibited reduced reactivity against all tested variants. The assay panel used substantiated the impact of anti-TNF therapy in IBD patients as to reduced strength, function, and breadth of the immune response to several SARS-CoV-2 variants. The immune response measured following the second vaccination was comparable to the antibody response observed in healthy individuals following the first vaccination. **Conclusion:** Variant-specific sVNTs and pVNTs have the potential to serve as valuable tools for evaluating the efficacy of adapted vaccines and to inform clinical interventions in the care of immunosuppressed patients. Anti-TNF-treated individuals with antibody levels below the optimized CMIA threshold should be considered for early booster vaccination and/or close immunological monitoring.

## 1. Introduction

The causative agent of coronavirus disease 2019 (COVID-19), the global pandemic of which began around November 2019, is the severe acute respiratory syndrome coronavirus 2 (SARS-CoV-2) [1,2]. In May 2023, the World Health Organization (WHO) formally announced the conclusion of the pandemic. Nevertheless, the persistent emergence of new virus variants continues to pose a challenge to healthcare systems globally. Whereas in the majority of cases the viral infection manifests as a mild to moderate illness, akin to the common cold, in a small number of cases the condition progresses to a severe form, characterized by acute respiratory distress syndrome (ARDS), and a high mortality rate [3,4,5,6,7,8]. Severe, critical disease courses have been observed to correlate with signs of hyperinflammation similar to classical cytokine storm syndromes [9,10,11]. In certain instances, this can result in respiratory failure, multiple organ failure, and ultimately, death. Notably, even after mild or moderate acute disease courses, persisting symptoms have been observed, a phenomenon referred to as Long- or Post-COVID-19 syndrome [12,13,14,15].

Immunosuppressive medications are employed in the treatment of inflammatory bowel diseases (IBDs) such as Crohn’s disease and ulcerative colitis [16,17]. A particularly elevated risk of developing severe infections is associated with the administration of anti-tumor necrosis factor (TNF) therapy [18]. In a previous study, we demonstrated that IBD patients under anti-TNF therapy exhibited a reduced antibody response compared to healthy controls following mRNA vaccination against SARS-CoV-2 [19,20,21]. Moreover, the administration of anti-TNF antibodies, such as infliximab and adalimumab, has been associated with a reduced extent and duration of antibody protection after infection and/or vaccination, and an elevated incidence of breakthrough infections [22,23]. Compared to the therapeutic anti-integrin antibody vedolizumab, which acts specifically in the intestine and causes less systemic immunosuppression, anti-TNF medication has been shown to result in a diminished antibody response after SARS-CoV-2 vaccination [24,25]. However, T cell responses to vaccines do not appear to be adversely affected [26,27]. In general, IBD patients appear to benefit from the SARS-CoV-2 vaccination to a similar extent as individuals not diagnosed with IBD, as reviewed by Ref. [28]. In most of the aforementioned studies, the assays for the assessment of binding antibody titers or neutralization capacity were exclusively based on the SARS-CoV-2 Wuhan-Hu-1 wild-type strain [1].

In the meantime, variants of the wild-type SARS-CoV-2 strain have developed due to mutations predominantly in the S1 subunit of the spike protein (S1) encompassing the viral receptor binding domain (RBD) [29]. Since its first description in November 2021, Omicron and its subvariants have spread globally [30]. Omicron and its descendants are considered more resistant to pre-existing antibodies, thus leading more frequently to re-infections or breakthrough infections in vaccinated individuals [31,32]. Hence, it is imperative to investigate the immune reaction of IBD patients who received Wuhan-Hu1-based vaccines against Omicron variants. While earlier studies comprising IBD patients mainly focused on concentrations of RBD-binding antibodies [33], their results seem to only partially reflect the functional antibody response to infections and, thus, warranted further examination of the neutralizing capacity of antibodies, e.g., by virus neutralization tests (VNTs). Moreover, assays have been developed to determine the neutralizing capacity of SARS-CoV-2 variant-specific antibodies, taking into account a diminished sensitivity exhibited by commercial tests based on wild-type strain sequences in regard to virus variants [34,35].

In this pilot study, we aimed to compare a small cohort of SARS-CoV-2 mRNA-vaccinated IBD patients under anti-TNF therapy with healthy individuals without previous SARS-CoV-2 infection regarding their functional antibody responses to several viral variants. Serum levels of RBD-binding antibodies were determined by chemiluminescence microparticle immunoassay (CMIA). Neutralization capacity was determined by a commercially available and by variant-specific in-house surrogate virus neutralization tests (sVNTs) and an in-house VSV-pseudotyped virus neutralization test (pVNT) based on SARS-CoV-2 wild-type strain Wuhan-Hu-1 [1], and variants Alpha, Beta, Gamma, Delta, Omicron BA.1, and Omicron BA.5.

## 2. Materials and Methods

### 2.1. Study Subjects and Samples

**Figure 1:**
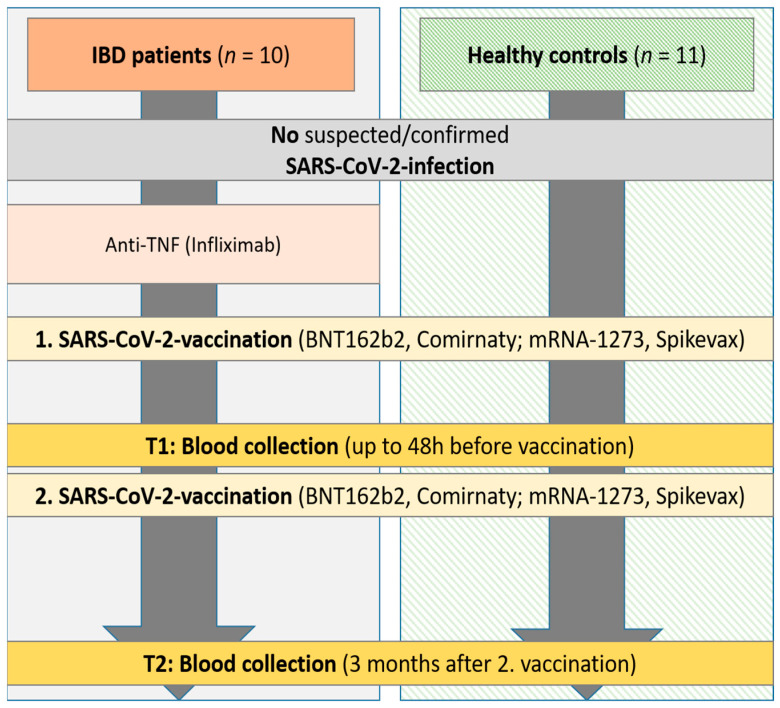
Study flow chart. Inclusion of IBD patients in 2021 on immunosuppressive medication (infliximab) and healthy control persons without suspected/confirmed SARS-CoV-2 infection. Blood collection up to 48 h before the second SARS-CoV-2 vaccination and 3 months after the second vaccination. IBD, inflammatory bowel disease; SARS-CoV-2, severe acute respiratory syndrome coronavirus 2.

Samples from IBD patients (*n* = 10) and matched healthy control persons (*n* = 11) without suspected or confirmed SARS-CoV-2 infection were collected at the IBD outpatient clinic of the Department of Gastroenterology, Hepatology, Endocrinology, and Clinical Infectiology, University Hospital Muenster, Germany, in the framework of a prospective study (January 2021–November 2021) [19]. The IBD patients included in this study received immunosuppressive therapy with anti-TNF antibodies (i.e., infliximab) for at least three months before undergoing their first mRNA immunization. In the IBD patients diagnosed with ulcerative colitis, the clinical Mayo score was determined at the time of blood collection, whereas in the Crohn’s disease patients, the Crohn’s disease activity index (CDAI score) was determined in each case. The vaccination of the study groups was performed with either BNT162b2 (*Comirnaty*, BioNTech SE/Pfizer, Mainz, Germany) or mRNA-1273 (*Spikevax*, Moderna Inc., Cambridge, MA, USA), both of which are monovalent, non-variant of concern (VOC) adapted mRNA vaccines. Patients were vaccinated solely according to their physicians’ recommendation, not for study purposes. The first blood sample was taken up to 48 h before the second vaccination (t1; Figure 1). Follow-up blood samples were taken three months (±7 days) after the second mRNA-based SARS-CoV-2 vaccination (t2). In IBD patients, samples of all participants were obtained at t1 and t2. In the control group, samples were collected from nine participants at time points t1 and t2. However, due to the lack of a tenth control having donated specimens at t1 and t2, samples were instead collected from two participants once at each of these time points, respectively. The local ethical committee (University Hospital Muenster 2021-039-f-S, 3 February 2021) approved this study.

### 2.2. Characterization of Sera with Commercial Assays

Quantification of total binding immunoglobulin G targeting the SARS-CoV-2 receptor binding domain (RBD) was performed by applying the chemiluminescence microparticle immunoassay (CMIA) *SARS-CoV-2 IgG II Quant* on an *Architect* platform (Abbott Diagnostics, Wiesbaden, Germany) according to the manufacturer’s manual as previously published [20,34]. Sera were characterized as positive if IgG values equaled to or exceeded the cut-off value set at 50 Arbitrary Units (AU)/mL, corresponding to 7.1 Binding Antibody Units (BAU) of the WHO International Standard for anti-SARS-CoV-2 immunoglobulin (human) (NIBSC code 20/136, WHO First International Standard for anti-SARS-CoV-2 immunoglobulin (human), National Institute for Biological Standards and Control, Hertfordshire, UK, 2020; https://www.nibsc.org/documents/ifu/20-136.pdf). The upper limit of the test’s linear detection range amounts to 40.000 AU/mL.

The RBD-binding inhibitory capacity of serum antibodies was assessed using the *cPass SARS-CoV-2 Neutralization Antibody Detection Kit* (GenScript Biotech, Leiden, The Netherlands) following the manufacturer’s instructions as previously described [20,34]. In brief, inhibition of the binding of horse-radish peroxidase-coupled RBD to the human cell receptor hACE2 immobilized on microtitre plates after incubation with human sera is measured [36]. The assay is based on RBD sequences derived from the wild-type (wt) SARS-CoV-2 Wuhan-Hu-1 strain. Values equal to or above the cut-off of 30% inhibition were considered positive. This surrogate virus neutralization assay has been demonstrated to correlate well with the gold standard, the conventional live-virus neutralization assays [37,38].

### 2.3. Characterization of the Neutralizing Capacity of Sera with In-House Assays

#### 2.3.1. VSV-Pseudotyped Virus Neutralization Assay

Neutralizing antibodies targeting the spike protein were quantified using a VSV-pseudotyped virus neutralization assay (pVNT) as described in Refs. [34,39]. Briefly, the GFP-Luc-expressing vesicular stomatitis virus lacking its surface glycoprotein (VSV ΔG/GFPLuc) was modified to present SARS-CoV-2 spike proteins based on the Wuhan-Hu-1 strain [1] as well as the Omicron BA.1 spike sequences, as outlined in [34]. Following pre-incubation of the patient and control sera diluted 1:20 with the pseudotyped virus, solutions were added to Vero E6 cell cultures at a multiplicity of infection (MOI) of 0.01 for 1 h, and GFP-expressing cells were quantified 16 h later, as published by Ref. [34]. Measurements were performed on quadruplicates, from which mean values were calculated. The neutralizing capacity of the sera was expressed as the reduction of the GFP signal (%) = (1 − GFP signal of the treated sample/GFP signal of the untreated sample) × 100%. According to Ref. [34], the cut-off value of the pVNT was set to ≥65% to achieve high specificity.

#### 2.3.2. Surrogate Virus Neutralization Assay

Secreted RBD fragments N-terminally tagged with nanoluciferase (NLuc, Promega, Walldorf, Germany) were used to assess receptor binding inhibition by sera on microtitre plates coated with recombinant hACE2, as described by Ref. [34]. Constructs containing RBD fragments spanning from residues 319 to 541 of the Wuhan-Hu-1 and Omicron BA.1 strains were generated as previously published [34]. Substitutions corresponding to the Alpha, Beta, Gamma, and Delta variants were generated and inserted into the Wuhan-Hu-1 RBD by site-directed mutagenesis. The RBD fragment of the Omicron BA.5 variant was amplified by PCR from the vector pcDNA3.1 SARS-CoV-2 S BA5 (custom-made by ThermoFisher, Schwerte, Germany) containing a synthetic S gene of variant Omicron BA.5 and inserted into plasmid pEN-secNL-RBD characterized in Ref. [34]. VOC-specific substitutions within the RBD-fragments are listed in Table S1. Sera were tested at a dilution of 1:20. Receptor binding inhibition was calculated as a reduction in the NLuc signal (%) = (1 − NLuc signal of the sample/NLuc signal of the untreated sample) × 100%. Cut-off values of wt RBD, Alpha RBD, Beta RBD, Gamma RBD, and Delta RBD sVNT were set to ≥25% inhibition and the cut-off value of BA.1 and BA.5 RBD sVNT to ≥50% inhibition.

### 2.4. Statistical Analysis

Data were assessed for normal distribution using the Kolmogorov–Smirnov test. For continuous variables, we reported medians with interquartile ranges and compared them using the Mann–Whitney U (Wilcoxon) test. For categorical variables, we reported absolute numbers and percentages and compared them using Chi-squared tests of association or Fisher’s exact tests. Kruskal–Wallis tests and Dunn’s and Friedman multiple comparison tests were conducted to compare more than two groups. All tests were two-tailed, and a *p*-value of <0.05 was considered to indicate a statistically significant difference. The diagnostic performance of assays was analyzed by receiver operating characteristic (ROC) curve analysis using the wt S pVNT as gold standard. Adapted cut-offs were calculated with Youden’s S statistics. Tukey boxplots are shown in the figures. All statistical analyses were performed using SPSS 26 (IBM, Chicago, IL, USA) and GraphPad Prism software (Version 8.0 for Microsoft, Version 10.4.1 for MacOS, GraphPad Software, La Jolla, CA, USA).

## 3. Results

### 3.1. Cohort Characteristics

Only patients and healthy control persons who did not have suspected or confirmed acute SARS-CoV-2 infection were included in this study. There were no significant differences in age or sex between the IBD patients and the healthy controls. The IBD patients had a median age of 50 years, and 40% were male. An oral mesalazine therapy was administered to 60% of the IBD patients, and an oral budesonide therapy was used in 10%. None of the patients were on oral prednisolone therapy. The most common co-morbidity among the patients was pre-existing cardiovascular disease (30%) (Table 1), while the controls had no known pre-existing conditions. Compared to the IBD patients, the healthy controls were more frequently vaccinated with the mRNA-1273 (Spikevax, Moderna) vaccine (0% of IBD patients, 77% of controls; *p* < 0.001) than with the BNT162b2 (Comirnaty, BioNTech/Pfizer) vaccine (100% of IBD patients, 23% of controls; *p* < 0.001). None of the patients or controls had died by the end of the study.

### 3.2. Effect of Anti-TNF Therapy on Antibody Levels

**Figure 2:**
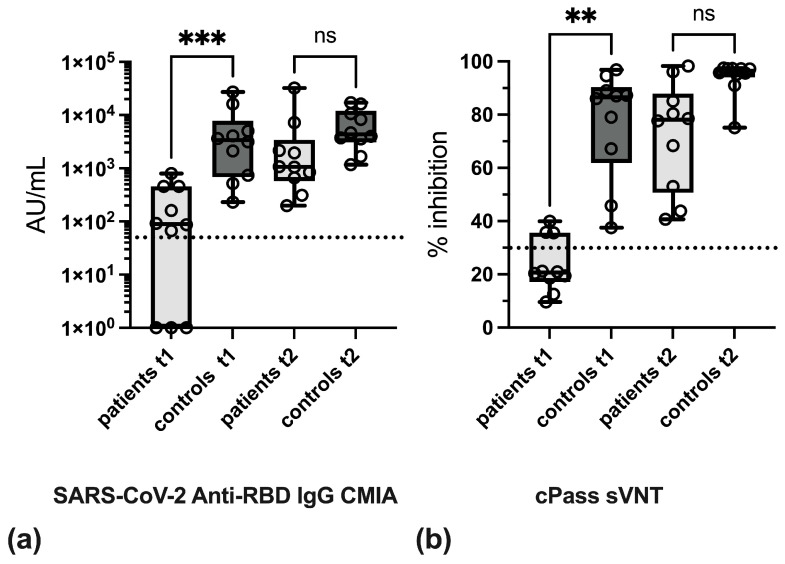
Results of routine serologic assays in IBD patients under anti-TNF therapy compared to healthy controls. (**a**) Total anti-RBD IgG levels were determined by SARS-CoV-2 IgG II Quant CMIA (Abbott, AU/mL) and (**b**) cPass sVNT (GenScript, % inhibition). Zero AU/mL values were set to one to enable the logarithmic representation. Boxes depict 25% to 75% percentiles, individual values are indicated, whiskers give min. and max. values, and the variable median is indicated by line. Significance was determined with Kruskal–Wallis test and Dunn’s multiple comparison test. ** *p* < 0.01, *** *p* < 0.001, ns: not significant. The cut-off values of the CMIA (≥50 AU/mL) and sVNT (30%) are indicated by dashed lines.

In order to demonstrate the quantitative effects of anti-TNF therapy on the formation of SARS-CoV-2-specific antibodies, the antibody levels in IBD patients and in the control group were compared in the respective test procedures after the first and second vaccinations. In the CMIA, antibody levels in IBD patients were found to be approximately 30-fold reduced at t1 as compared to the control group, whereas at t2, these differences no longer achieved statistical significance (Figure 2a, Table S2). Likewise, in the cPass sVNT, levels of inhibitory antibodies were significantly diminished in IBD patients at t1 (Figure 2b), while at t2, differences in inhibitory antibody levels between IBD patients and controls were no longer statistically significant.

**Figure 3:**
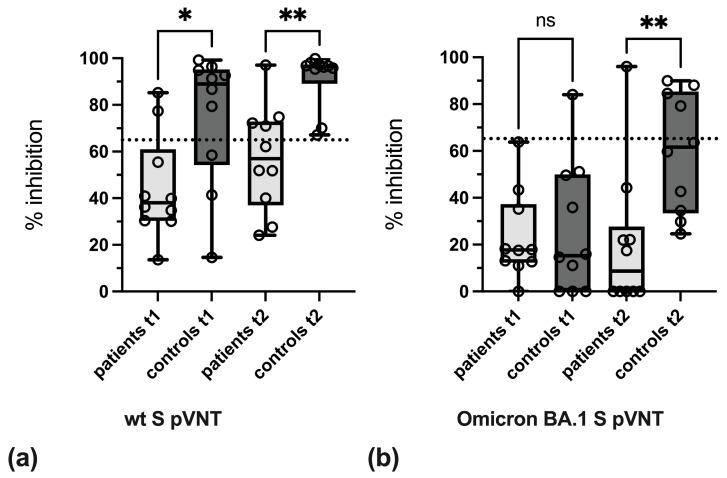
Determination of neutralizing antibodies against (**a**) wild-type S and (**b**) Omicron BA.1 S by pVNT. Boxes depict 25% to 75% percentiles, min. and max. values are given by whiskers, individual values are shown, the variable median is indicated by line. The significance of differences was determined by Kruskal–Wallis test and Dunn’s multiple comparison test. * *p* < 0.05, ** *p* < 0.01, ns: not significant. The cut-off value of the pVNT (≥65% inhibition) is indicated by dashed lines.

The quantitative analysis of pVNT results showed that levels of neutralizing antibodies against wt S were significantly lower in patient sera at time points t1 and t2. Due to the poor reactivity of sera in the BA.1 S pVNT at t1, differences between IBD patients and controls were not significant. At t2, levels of neutralizing antibodies against BA.1 S were significantly higher in control persons as compared to IBD patients (Figure 3a,b, Table S2).

**Figure 4:**
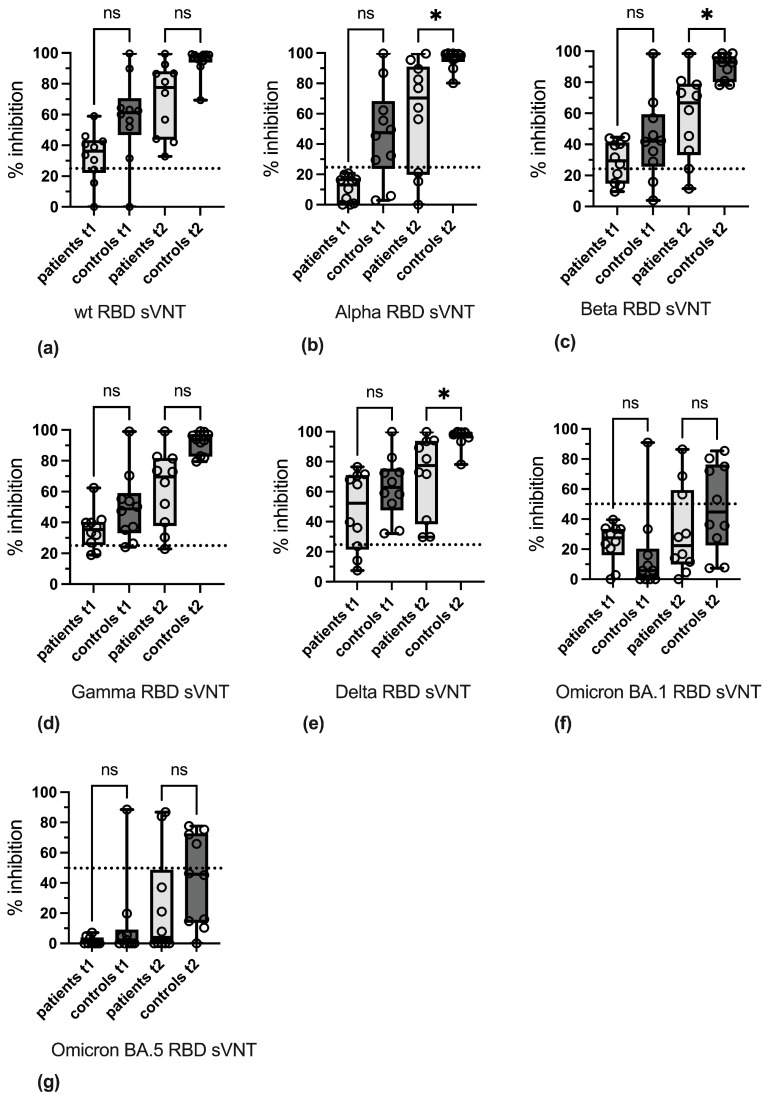
Determination of variant-specific inhibitory antibodies by in-house sVNT. Reactivity of patient and control sera with (**a**) wild-type RBD, (**b**) Alpha RBD, (**c**) Beta RBD, (**d**) Gamma RBD, (**e**) Delta RBD, (**f**) BA.1 Omicron RBD, and (**g**) BA.5 Omicron RBD is shown. Boxes depict 25 to 75% percentiles, individual values are indicated, whiskers give min. and max. values, and the variable median is indicated by line. Significance was determined with Kruskal–Wallis test and Dunn’s multiple comparison test. * *p* < 0.05, ns: not significant. The cut-off values of the sVNTs are indicated by dashed lines.

Analysis of antibody reactivity by in-house sVNT also indicated that overall antibody levels in patient sera at t1 and t2 were decreased as compared to controls. These differences were statistically significant at t2 in the Alpha RBD sVNT, Beta RBD sVNT, and Delta RBD sVNT (Figure 4).

In addition, antibody levels after the first and second vaccinations in IBD patients and controls were compared pairwise. Significant rises in antibody levels were observed in IBD patients in CMIA, cPass sVNT, wt RBD sVNT, Alpha RBD sVNT, Beta RBD sVNT, and Gamma RBD sVNT, and in the control group in the cPass sVNT, BA.1 S pVNT, and in all in-house sVNTs (Table S2).

### 3.3. Variant-Specific Differences in Reactivity

**Figure 5:**
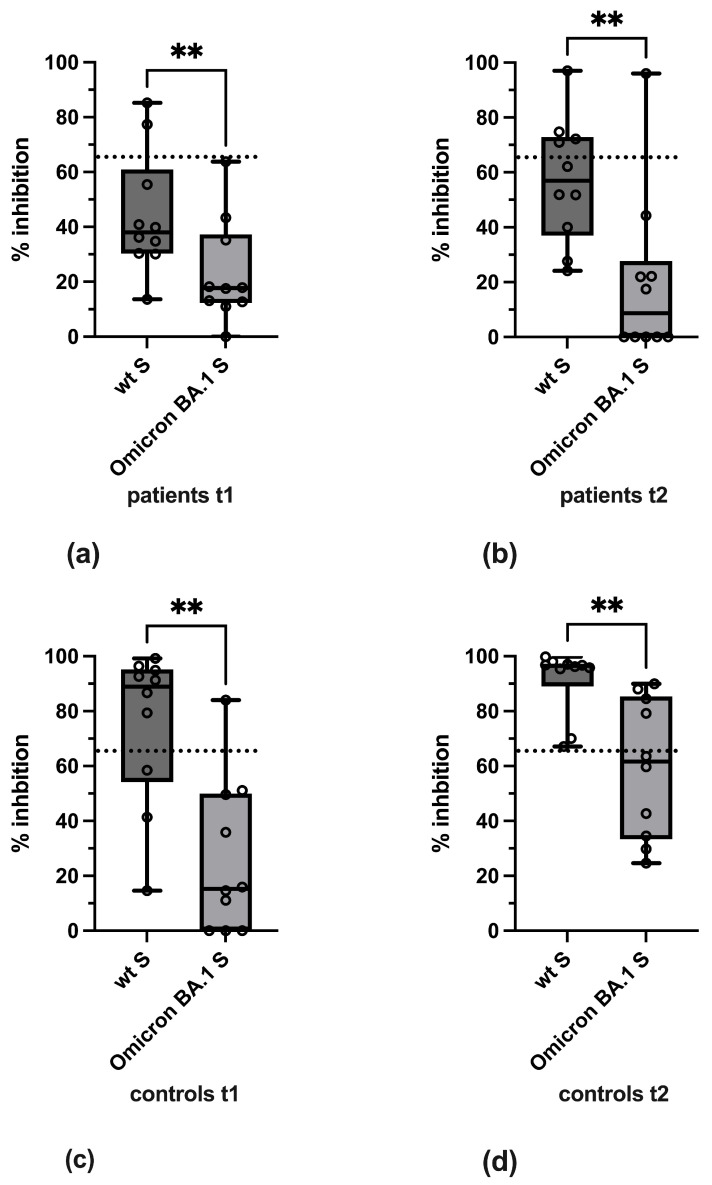
Pairwise comparison of neutralizing antibodies against wild-type S and BA.1 Omicron S by pVNT for (**a**) IBD patients at t1, (**b**) IBD patients at t2, (**c**) controls at t1, and (**d**) controls at t2. Boxes depict 25% to 75% percentiles, min. and max. values are given by whiskers, individual values are shown, the variable median is indicated by line. The significance of differences was determined by Wilcoxon test. ** *p* < 0.01. The cut-off value of the pVNT (≥65% inhibition) is indicated by dashed lines.

To analyze variant-specific differences in reactivity, a pairwise comparison of antibody levels against the wild-type and variant-specific forms of S and RBD used in the pVNT and sVNT, respectively, was performed in both groups at both time points. This showed that in both IBD patients and controls, levels of neutralizing antibodies against BA.1 Omicron at t1 and t2 were significantly lower as compared to wild-type S (Figure 5).

**Figure 6:**
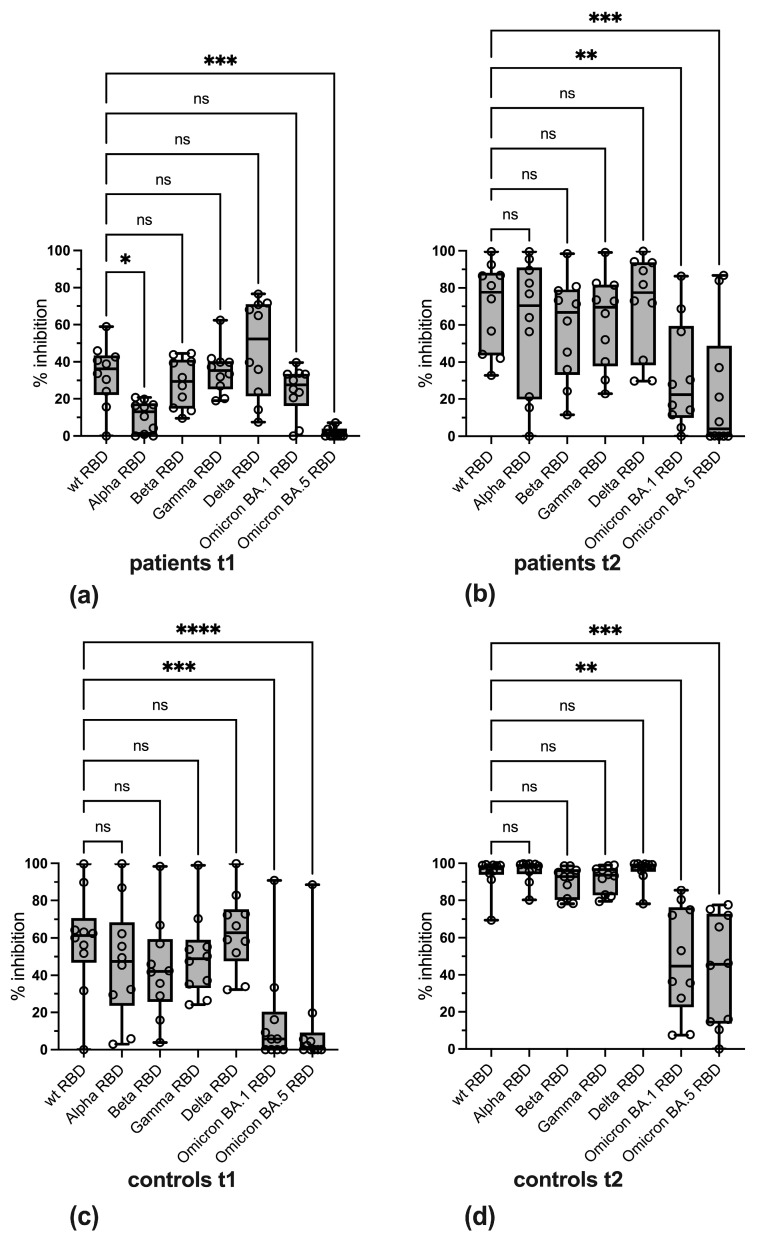
Pairwise comparison of wild-type with variant-specific inhibitory antibodies in the sVNT for (**a**) IBD patients at t1, (**b**) IBD patients at t2, (**c**) controls at t1, and (**d**) controls at t2. Boxes depict 25% to 75% percentiles, min. and max. values are given by whiskers, individual values are shown, the variable median is indicated by line. The significance of differences was determined by Dunn’s multiple comparison test and Friedman test. * *p* < 0.05, ** *p* < 0.01, *** *p* < 0.001, **** *p* < 0.0001, ns: not significant.

Pairwise comparison of wt and variant-specific reactivity in the in-house sVNTs revealed that in IBD patients, levels of inhibitory antibodies against the Alpha RBD and the BA.5 RBD were significantly lower at t1, whereas at t2, a significant decrease in antibody levels against the BA.1 RBD and BA.5 RBD was observed, with median levels below the cut-off value (Figure 6a,b). In control sera, levels of inhibitory antibodies against the BA.1 RBD and the BA.5 RBD were significantly reduced at both time points (Figure 6c,d).

### 3.4. Qualitative Evaluation of SARS-CoV-2-Specific Antibody Reactivity

**Figure 7:**
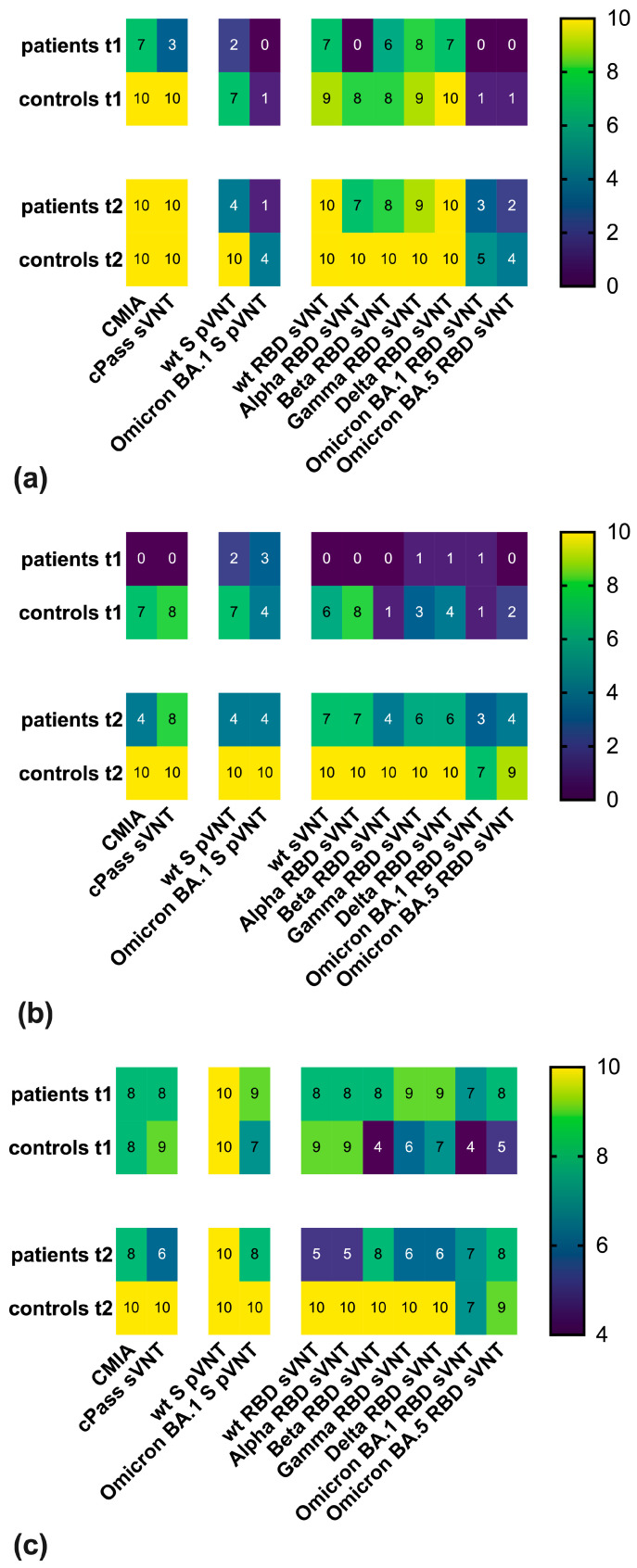
Qualitative differences in reactivity of IBD patients and the control group. The heatmaps display the number of sera (out of 10) reactive (**a**,**b**) in the respective assays at time points t1 and t2. In (**a**), the following cut-off values were used to categorize reactive and non-reactive sera: pVNT ≥ 65% inhibition, CMIA ≥ 50 AU/mL, cPass sVNT ≥ 30% inhibition; wt, Alpha, Beta, Gamma, Delta RBD in-house sVNTs ≥ 25% inhibition; BA.1 RBD sVNT and BA.5 RBD sVNT ≥ 50% inhibition. In (**b**), adapted cut-off values were used to differentiate between reactive and non-reactive sera: CMIA ≥ 1126 AU/mL, cPass sVNT: ≥49.4% inhibition, BA.1 S pVNT ≥ 20.09% inhibition, wt RBD sVNT ≥ 59.55% inhibition, Alpha RBD sVNT ≥ 25.35% inhibition, Beta RBD sVNT ≥ 72.27% inhibition, Gamma RBD sVNT ≥ 54.6% inhibition, Delta RBD sVNT ≥ 72.07% inhibition, BA.1 S RBD sVNT ≥ 34.73% inhibition, and BA.5 S RBD sVNT ≥ 9.095% inhibition. (**c**) The heat map displays the number of sera yielding results concordant with those of the wt S pVNT as the gold standard (true positive plus true negative results).

In a further step, it was investigated whether qualitative classification of sera into reactive and non-reactive categories in the respective assays was sufficient to characterize the antibody response in patient and control sera and to highlight differences between both groups. To this end, cut-off values as suggested by the assays’ manufacturers were applied in the CMIA (≥50 AU/mL) and cPass sVNT (≥30% inhibition). Cut-off values published earlier [34,40] were used for wt S pVNT (≥65% inhibition) and wt RBD sVNT (≥25% inhibition). Moreover, the present study investigated qualitative reactivity in assays employing variant-specific antigens. A cut-off value of ≥65% inhibition was used for BA.1 S pVNT [40]. Cut-off values of Alpha RBD, Beta RBD, Gamma RBD, and Delta RBD sVNTs were set to ≥25% inhibition and the cut-off value of BA.1 and BA.5 RBD sVNTs to ≥50% inhibition. The application of these cut-off values to differentiate between reactive and non-reactive categories revealed a lower vaccination-induced antibody response in IBD patients as compared to the control group both at t1 and, to a lesser extent, at t2 (Figure 7a).

In general, the number of sera-reactive in the Omicron-specific assays was found to be reduced in both study groups. A comparison of the BA.1 S pVNT with the BA.1 RBD and BA.5 RBD sVNTs showed that the results of these assays correlated. Thus, the single patient serum reactive in BA.1 S pVNT at t2 exhibited the strongest signal in the BA.1 and BA.5 RBD sVNTs. In addition, at t1, a single control serum was reactive in BA.1 S pVNT and the BA.1 and BA.5 RBD sVNTs. Furthermore, all four control sera reactive in the BA.1 S pVNT at t2 also displayed reactivity in the BA.1 and BA.5 RBD sVNTs.

Particularly noticeable was the poor reactivity of patient sera at t1 and t2 in the wt S pVNT as well as in the cPass sVNT and Alpha RBD sVNT at t1. In contrast, most patient sera were classified as reactive in the CMIA and the Gamma RBD and Delta RBD sVNTs already at t1. The poor agreement of most assays with the qualitative results of the wt S pVNT in IBD patients prompted us to re-evaluate the cut-off values used in the assay panel. The diagnostic performance of the other assays was assessed by ROC curve analysis, with reactivity in the wt S pVNT (cut-off ≥ 65% inhibition at a serum dilution of 1:20) serving as the gold standard for the presence or absence of neutralizing antibodies in the 40 patient and control sera included in this study (Figure S1). Although none of the assays showed complete concordance with the wt S pVNT, the results of wt S pVNT strongly correlated with the results of the BA.1 S pVNT, CMIA, cPass sVNT, and in-house sVNTs employing non-Omicron RBDs. In contrast, the BA.1 RBD and BA.5 RBD sVNTs showed no or only negligible correlation with the wt S pVNT. To improve the level of agreement between the wt S pVNT and the remaining assays, data of ROC curves were analyzed by Youden’s J statistics to define adapted cut-off values. When determining the relative optima, equal weight was attributed to increases in sensitivity and specificity (Table 2).

With the exception of the BA.1 S pVNT and the BA.1 RBD and BA.5 RBD sVNTs, the cut-off values of most of the assays had to be raised to achieve a better agreement with the wt S pVNT. Overall, these adjustments mainly resulted in a reduced number of patient sera reactive at t1 and t2 and of control sera reactive at t1 (Figure 7b). Concerning variant-specific sVNTs, missing to poor reactivity of patient sera was observed at t1. In addition, reactivity of control sera at t1 in the Beta RBD, BA.1 RBD, and BA.5 RBD sVNTs was strongly reduced. Using these adapted cut-off values, the number of positive and negative results concordant with the result of wt S pVNT was determined. This analysis showed that by adjusting the cut-off values, the CMIA and the cPass sVNT exhibited a high concordance, e.g., 87.5% and 82.5%, respectively, with the wt S pVNT in patient and control sera at t1 and t2 (Figure 7c). The utilization of adapted cut-offs also revealed a good agreement between the results of the wt S pVNT and the results of some assays employing variant-specific antigens, such as the BA.1 S pVNT, and, albeit to a lesser extent, the Alpha RBD and Delta RBD sVNTs. In contrast, a reduced reactivity was evident in the Beta RBD, Gamma RBD, BA.1 S RBD, and BA.5 RBD sVNTs in patient sera at t2 and in control sera at t1 (Figure 7b, c).

## 4. Discussion

Total antibody levels against SARS-CoV-2 spike exhibit a strong positive correlation with the titers of neutralizing antibodies, which represent a correlate of protection (CoP). Thus, the determination of total antibody levels enables the prediction of anticipated antibody-mediated antiviral effects [41,42,43,44,45,46,47,48,49]. In an effort to alleviate the comparison of antibody reactivity in fully automated routine serology tests, the S-specific total antibody levels have been normalized to BAU/mL. Nonetheless, significant differences between various assays have been observed, underscoring the necessity for further standardization of SARS-CoV-2 serology [50,51,52,53,54,55,56]. At present, the definition of an antibody limit titer above which a protective effect against infection can be expected at the individual level, for example, is therefore considered virtually impossible to achieve using routine serology mass throughput methods [57,58].

Hence, a more attainable interim goal could be to initially evaluate the humoral immune response in risk groups with regard to total antibody levels, inhibitory antibody levels and, if necessary, the variant specificity of antibodies using methodologically less complex test procedures. The adjustment of cut-off values [59] and the subsequent categorization of tested individuals as reactive or non-reactive is expected to facilitate the identification of individuals for whom the formation of neutralizing antibodies is uncertain. These individuals, designated as “non-reactive,” may necessitate additional interventions, such as further vaccination or adjustment of immunosuppressive therapy. In the present study, this question was addressed in a small cohort of anti-TNF-treated IBD patients and healthy individuals.

When sera collected after the first and second vaccination were categorized into reactive and non-reactive samples using wt S pVNT, a highly significant correlation between the result of the pVNT and the total anti-RBD IgG levels measured by CMIA was found in our cohort, in agreement with numerous previous studies [60]. Our results show that in a mixed cohort of immunocompromised patients and controls, raising the cut-off value of the CMIA, which is designed by the manufacturer for high sensitivity, from 50 AU/mL to around 1100 AU/mL or 150 BAU/mL, enables the identification of sera reactive in the pVNT with a sensitivity of approximately 83% and specificity of approximately 88%. This corresponds well to the results of a previous study in which we showed that in a larger cohort of healthy vaccinees, by raising the cut-off value of the CMIA to about 1700 AU/mL or 240 BAU/mL, the identification of sera from healthy vaccinees containing neutralizing antibodies against SARS-CoV-2 after a second vaccination is feasible with a specificity of 97% and a sensitivity of 96% [34]. The definition of different limiting titers in the CMIA and thus achievable sensitivities and specificities in both studies is most likely explained by different sampling time points and the testing of a mixed cohort of immunosuppressed and healthy individuals in the current study. With regard to the measuring range, the CMIA appears to cover a wide range of antibody concentrations [61].

In routine clinical practice, the capacity to identify individuals who have received anti-TNF treatment in the absence of neutralizing antibodies is of paramount importance, as such medication is associated with a diminished likelihood of neutralizing antibody formation. Consequently, additional measures may be required to mitigate the risk of reinfection, including repeated vaccinations or adjustments to immunosuppressive therapy [25,61]. The population investigated in this study is challenging due to the presence of immunosuppressed subjects beneath healthy controls, as well as samples obtained before and after the first and second vaccinations. A correct diagnosis was achieved in 34 out of 40 cases, with the cut-off value of the CMIA being raised to approximately 1100 AU/mL. Nevertheless, it is essential to recognize that establishing a cut-off value is a precarious endeavor, requiring a meticulous balance between attaining optimal sensitivity and specificity. This phenomenon leads to an underestimation of the formation of neutralizing antibodies in anti-TNF-treated patients after the first vaccination, as determined by the CMIA. Consequently, subsequent to the adjustment of the threshold values, two patients with confirmed neutralizing antibodies at t1 and a single patient at t2 were no longer identified as positive in the CMIA. The observed discrepancy in reactivity between the CMIA and the pVNT can potentially be attributed to the inability of the CMIA to detect NTD-specific antibodies or the isotype-independent reactivity of neutralizing antibodies in the pVNT.

The adjusted CMIA cut-off value of approximately 1100 AU/mL demonstrated a diagnostic performance sufficient to stratify patients in routine clinical settings, enabling clinicians to identify IBD patients under anti-TNF therapy who are unlikely to have developed neutralizing antibodies and who may therefore benefit from additional protective strategies. Based on our findings, we recommend that anti-TNF-treated individuals with antibody levels below the optimized CMIA threshold should be considered for early booster vaccination and/or close immunological monitoring. This approach may help reduce the risk of SARS-CoV-2 breakthrough infections in this vulnerable subgroup.

Tested as an alternative routine method, the cPass sVNT exhibited a high degree of concordance with the results of the pVNT when the cut-off value was elevated from 30% to approximately 50% inhibition (33 of 40 cases with matching results). Previous studies have highlighted a significant correlation between the cPass sVNT and conventional VNTs, as well as the possibility of calibrating the cPass sVNT result to IU/mL [38,51,59]. Notably, despite its capacity to detect functional antibodies that inhibit RBD binding to ACE2, the cPass sVNT did not exhibit superior diagnostic performance compared to CMIA in the population evaluated in this study. Given that the control sera had shown saturation of antibody-induced inhibition already at t2, the determination of end titers using the cPass sVNT has the potential to improve diagnostic performance.

A further parameter for determining the quality of the humoral immune response against SARS-CoV-2 is the breadth of antibody reactivity, which was assessed using pVNT and sVNT in the cohort studied here. As anticipated based on the presence of multiple immune escape mutations in the Omicron spike protein [62,63], there was a marked reduction observed in the reactivity of patient and control sera in the Omicron BA.1 S pVNT at both collection time points. The present findings underscore the necessity for a third vaccination, particularly within the cohort of immunocompromised patients [64]. Despite the considerably diminished overall levels, the results of the BA.1 S pVNT demonstrated a remarkably strong correlation with the results of the wt S pVNT. On the one hand, this finding confirms that Omicron-reactive antibodies are already being formed, albeit to a lesser extent, upon immunization with the wt spike. On the other hand, the pVNT also detects S-reactive neutralizing antibodies binding outside the RBD, the antigen utilized for the CMIA and the cPass sVNT, e.g., in the NTD area.

In-house sVNTs developed in our research group [34] were used as a further method to determine the breadth of the antibody response. The sVNT has proven to be a valuable tool for the detection of variant-specific antibodies, and its results correlate with those of the VNT [65,66]. Labeling the RBD with Nanoluc renders the test design employed by our laboratory well suited to the production and testing of variant-specific forms of the RBD.

This sVNT design enabled the substantiation of the pronounced immune escape function of the numerous mutations present in the RBD of the tested Omicron variants BA.1 and BA.5. This finding aligns with the conclusions of prior studies, which likewise demonstrated the efficacy of sVNTs in detecting VOC-specific inhibitory antibodies [67]. When testing the pre-Omicron variants Alpha, Beta, Gamma, and Delta, the lack of reactivity of the patient sera post-initial vaccination in the Alpha RBD sVNT was particularly striking. The RBD of Alpha does not contain any typical escape mutations; however, it has a significantly increased affinity for ACE2 and fitness due to the N501Y mutation [68,69,70]. Therefore, the lack of reactivity of the patient sera after the first vaccination might indicate reduced antibody maturation and the formation of antibodies with a lower avidity due to anti-TNF therapy [71]. A comparable reduction in reactivity to that observed in the Alpha RBD sVNT in patient sera at t1 was not detected in patients and controls when the Beta, Gamma, and Delta sVNTs were employed. Given that the Omicron RBD has an affinity for ACE2 analogous to that of the wild-type RBD [69], the observed decrease in reactivity in the sVNT can be attributed to the multiple escape mutations.

By adjusting the cut-off values of the in-house sVNTs, it was also possible to achieve better overall agreement with the results of the wt S pVNT. In the qualitative evaluation, a correct diagnosis with regard to the pVNT as the gold standard was achieved in over 80% of cases. Furthermore, qualitative disparities in reactivity with the VOCs Beta, Gamma, Delta, and the two Omicron variants tested became more evident. With regard to variant-specific reactivity, patient sera at t2 exhibited a reaction pattern that was analogous to that of control sera at t1.

The employment of variant-adapted vaccines has been demonstrated to provide superior protection against the currently circulating Omicron strains [58,72]. Given the capacity of the in-house assays outlined here to discern variant-specific antibody reactions, a more comprehensive assessment of the protective efficacy of antibodies can be anticipated, superseding the capabilities of the prevailing routine tests that primarily employ antigens related to the Wuhan virus strain [73]. The inclusion of variant-specific sVNTs in the present study revealed differences in antibody reactivity not captured by assays based solely on the ancestral spike protein. These findings support the clinical utility of variant-adapted serological testing to more accurately assess vaccine-induced immunity and guide decision-making in immunocompromised patients.

The data presented here support the implementation of semi-quantitative antibody monitoring as part of a personalized risk assessment strategy in immunosuppressed individuals. While functional neutralization tests may not be feasible in all settings, optimized serological assays could serve as a surrogate to identify patients at higher risk and tailor intervention strategies accordingly. Our findings contribute to the refinement of serological monitoring protocols in high-risk patient populations and emphasize the value of incorporating quantitative antibody metrics into clinical workflows. Although a universally protective antibody threshold remains elusive, the optimized use of available serological assays can support timely, evidence-based interventions in the care of immunosuppressed patients.

The presence of antinuclear antibodies (ANAs) has been observed in approximately 50–80% of patients diagnosed with autoimmune diseases [74,75,76]. In the patient population diagnosed with inflammatory bowel disease, the prevalence of ANAs is approximately 14%, while seroconversion occurs in approximately 28% of individuals undergoing immunosuppressive anti-TNF therapy [74]. In patients with COVID-19, a broad range of autoantibodies, including ANA, anti-Ro/SSA, rheumatoid factor, lupus anticoagulant, and antibodies against type I interferons, have been identified [77], and an increased risk of developing autoimmune diseases such as rheumatoid arthritis, pemphigus, or pemphigoid has been reported [78]. Whether COVID-19 infection directly influences the course of autoimmune diseases in IBD patients remains unclear. However, most studies suggest that COVID-19 does not negatively affect the clinical course of IBD [79]. Although immunosuppressed patients may have an increased risk of severe COVID-19 outcomes, anti-TNF therapy in IBD patients has been associated with a reduced risk of critical disease progression [80]. This might be attributed to a modulatory effect on autoimmune responses during or after infection. Notably, prior SARS-CoV-2 vaccination has been shown to markedly reduce the risk of developing autoimmune diseases [78], underscoring the importance of vaccination in preventing long-term post-COVID-19 complications. Consequently, subsequent investigation into the humoral immunity against SARS-CoV-2 should encompass ANA screening in IBD patients, a component that was not included in the present study.

The limitations of the present study are primarily attributable to its modest sample size and its exclusive assessment of antibody reactivities following the first and second vaccinations. Furthermore, patients immunized with Omicron-matched vaccines or who have experienced an infection with an Omicron variant were not included. Consequently, it is challenging to establish limit titers for the qualitative evaluation of results, to make statements about the long-term course of antibody levels, and to assess the Omicron-specific antibody reactivity.

In conclusion, the test panel employed in the present study unequivocally substantiates the impact of anti-TNF therapy in patients suffering from IBD in terms of reduced strength, function, and the breadth of the immune response. The immune response measured after the second vaccination is comparable to the antibody response observed in the control group following the first vaccination.

## Figures and Tables

**Table 1:** Cohort characteristics of IBD patients. IBD: inflammatory bowel disease; IQR: interquartile range; BMI: body mass index; SARS-CoV-2: severe acute respiratory syndrome coronavirus 2; CDAI score: Crohn’s disease activity index, p.o.: per os; supp.: suppositories.

Patients		Infliximab (n = 10)	Healthy Controls (n = 11)	*p*-Value
**Patient characteristics**	Age, years median (IQR)	50 (31–65)	35 (23–66)	0.285
	Sex, male (%)	4 (40)	4 (36)	0.863
**SARS-CoV-2 vaccine**	Comirnaty BNT162b2 (%)	10 (100)	4 (23)	
	Spikevax mRNA-1273 (%)	0 (0)	7 (77)	
**IBD**	Crohn’s disease (%)	8 (80)	0 (0)	
	Ulcerative colitis (%)	2 (20)	0 (0)	
**Medication**	Prednisolone p.o. (%)	0 (0)	0 (0)	
	Budesonide p.o. (%)	1 (10)	0 (0)	
	Budesonide supp. (%)	0 (0)	0 (0)	
	Mesalazine p.o. (%)	6 (60)	0 (0)	
	Mesalazine supp. (%)	0 (0)	0 (0)	
**Pre-existing conditions**	Cardiovascular disease (%)	3 (30)	0 (0)	0.251
	Respiratory disease (%)	0 (0)	0 (0)	1.000
	Kidney insufficiency (%)	0 (0)	0 (0)	1.000
	Metastatic neoplasm (%)	0 (0)	0 (0)	1.000
	Diabetes (%)	0 (0)	0 (0)	1.000
	Hematologic malignancy (%)	0 (0)	0 (0)	1.000

**Table 2:** Diagnostic performance of assays as compared to wt S pVNT as the gold standard.

Assay	Area ^1^	*p* Value ^1^	Adapted Cut-Off Value ^1^	Sensitivity (%) at Adapted Cut-Off Value ^1^	Specificity (%) at Adapted Cut-Off Value ^1^
BA.1 S pVNT	0.8670	<0.0001	≥20.09%	82.61	88.24
Anti-RBD CMIA	0.8619	0.0001	≥1126 AU/mL	82.61	88.24
cPass sVNT	0.8542	0.0002	≥49.40%	91.3	70.59
wt RBD sVNT	0.8338	0.0004	≥59.55%	78.26	76.47
Alpha RBD sVNT	0.8414	0.0003	≥25.35%	86.96	70.59
Beta RBD sVNT	0.8159	0.0007	≥72.27%	60.87	94.12
Gamma RBD sVNT	0.8338	0.0004	≥54.60%	73.91	82.35
Delta RBD sVNT	0.8235	0.0005	≥72.07%	78.26	82.35
BA.1 RBD sVNT	0.6176	0.2082	≥34.73%	43.48	88.24
BA.5 RBD sVNT	0.7187	0.0193	≥9.095%	60.87	94.12

^1^ Diagnostic performance of assays in IBD patient and control sera (n = 40) was determined by ROC curve analysis, ≥65% inhibition in wt S pVNT at a serum dilution of 1:20 was used as cut-off value to discriminate between reactive and non-reactive sera, cut-off values for optimal performance of assays were determined by Youden’s J statistic.

## Data Availability

All experimental data relevant to the study have been included in the article. The raw data supporting the conclusions of this article will be made available by the authors on request. Patients’ data are not publicly available due to privacy/ethical restrictions. Further data are available on reasonable request.

## Supplementary Materials

The following supporting information can be downloaded at: https://www.mdpi.com/article/10.3390/vaccines13060595/s1, Table S1: List of VOC-specific substitutions introduced into NLuc-tagged RBD-fragments; Table S2: Differences in antibody levels in the study cohort; Figure S1: Analysis of sensitivity and specificity of assays by ROC curve analysis using the results of the wt S pVNT as gold standard.

## Abbreviations

**Abbreviation**	**Definition**
AU	arbitrary units
BAU	binding antibody units
CMIA	chemiluminescence microparticle assay
hACE2	human angiotensin-converting enzyme 2
NLuc	nanoluciferase
pVNT	VSV-pseudotyped virus neutralization assay
RBD	receptor-binding domain
ROC	receiver operating characteristic
SARS-CoV-2	severe acute respiratory syndrome coronavirus 2
sVNT	surrogate virus neutralization test
TNF	tumor necrosis factor
