# Supplementary material for: SARS-CoV-2 Variant-Specific Antibodies in Vaccinated Inflammatory Bowel Disease Patients
Source: Vaccines (Basel). 2025 May 30;13(6):595. doi: 10.3390/vaccines13060595 (PMC12197397; doi:10.3390/vaccines13060595)
Supplement: Supplementary file 1 [file vaccines-13-00595-s001.zip › vaccines-3630701-Supplementary.pdf]

Table S1. List of VOC-specific substitutions introduced into NanoLuc-tagged RBD fragments.

Variant form of RBD	Substitution(s)
Alpha RBD	N501>Y
Beta RBD	K417>N, E484>K, N501>Y
Gamma RBD	K417>T, E484>K, N501>Y
Delta RBD	L452>R, T478>K
Omicron BA.1 RBD	G339>D, S371>L, S373>P, S375>F, K417>N, N440>K, G446>S, S477>N, T478>K, E484>A, Q493>R, G496>S, Q498>R, N501>Y, Y505>H
Omicron BA.5 RBD	G339>D, S371>F, S373>P, S375>F, T376>A, D405>N, R408>S, K417>N, N440>K, L452>R, S477>N, T478>K, E484>A, F486>V, Q498>R, N501>Y, Y505>H

Table S2. Differences in antibody levels in the study cohort.

Assay		Antigen			Patients (Infliximab)	Healthy controls	p-value (Kruskal-Wallis test)
Anti-RBD IgG CMIA	AU/mL	wt RBD	t1	mean	212	6283	0,0020
				median	90	3385	
				IQR	1-457	685-7807	
			t2	mean	4796	7122	0,2885
				median	1068	4342	
				IQR	575-3438	3081-12037	
				p-value[1]	0,0039	0,1934	
cPass SARS-CoV-2	inhibition (%)	wt RBD	t1	mean	23	77	0,0046
				median	21	87	
				IQR	17-36	62-90	
			t2	mean	72	94	0,1415
				median	78	97	
				IQR	51-88	94-97	
				p-value[1]	0,0020	0,0020	
pVNT SARS-CoV-2 Inhibition (%)	inhibition (%)	wt S	t1	mean	44	75	0,0319
				median	38	89	
				IQR	30-61	54-95	
			t2	mean	57	91	0,0069
				median	57	96	
				IQR	37-73	89-97	
				p-value[1]	>0,9999	0,5529	
	inhibition (%)	Omicron BA.1 S	t1	mean	23	26	>0,9999
				median	18	15	
				IQR	12-37	0-50	
			t2	mean	20	60	0,0044
				median	9	62	
				IQR	0-28	33-85	
				p-value[1]	0,6953	0,0098	
In-house sVNT	inhibition (%)	wt RBD	t1	mean	33	58	0,0933
				median	36	61	
				IQR	22-43	47-71	
			t2	mean	70	94	0,0741
				median	78	98	
				IQR	44-88	94-99	
				p-value[1]	0,0098	0,0039	
	inhibition (%)	Alpha RBD	t1	mean	10	47	0,0776
				median	13	47	
				IQR	0.6-18	24-68	
			t2	mean	60	96	0,0480
				median	70	99	
				IQR	20-91	94-100	
				p-value[1]	0,0059	0,0039	

	inhibition (%)	Beta RBD	t1	mean	29	44	0,4713
				median	29	42	
				IQR	15-41	26-59	
			t2	mean	58	90	0,0434
				median	67	93	
				IQR	33-79	80-97	
				p-value[1]	0,0137	0,0039	
	inhibition (%)	Gamma RBD	t1	mean	35	50	0,4275
				median	35	49	
				IQR	25-40	33-59	
			t2	mean	62	91	0,0505
				median	69	94	
				IQR	38-82	83-97	
				p-value[1]	0,0488	0,0039	
	inhibition (%)	Delta RBD	t1	mean	47	63	0,5022
				median	52	63	
				IQR	21-71	48-75	
			t2	mean	70	96	0,0434
				median	77	99	
				IQR	38-94	95-100	
				p-value[1]	0,1602	0,0039	
	inhibition (%)	Omicron BA.1 RBD	t1	mean	24	16	0,2961
				median	28	6	
				IQR	16-34	0-20	
			t2	mean	32	48	0,3415
				median	22	45	
				IQR	10-59	22-76	
				p-value[1]	0,6953	0,0098	
	inhibition (%)	Omicron BA.5 RBD	t1	mean	2	11	0,6787
				median	0	1	
				IQR	0-4	0-9	
			t2	mean	24	42	0,2226
				median	4	46	
				IQR	0-49	14-73	
				p-value[1]	0,0781	0,0273	

[1] Significance of differences between t1 and t2 within groups was calculated using Wilcoxon matched-pairs signed rank test.

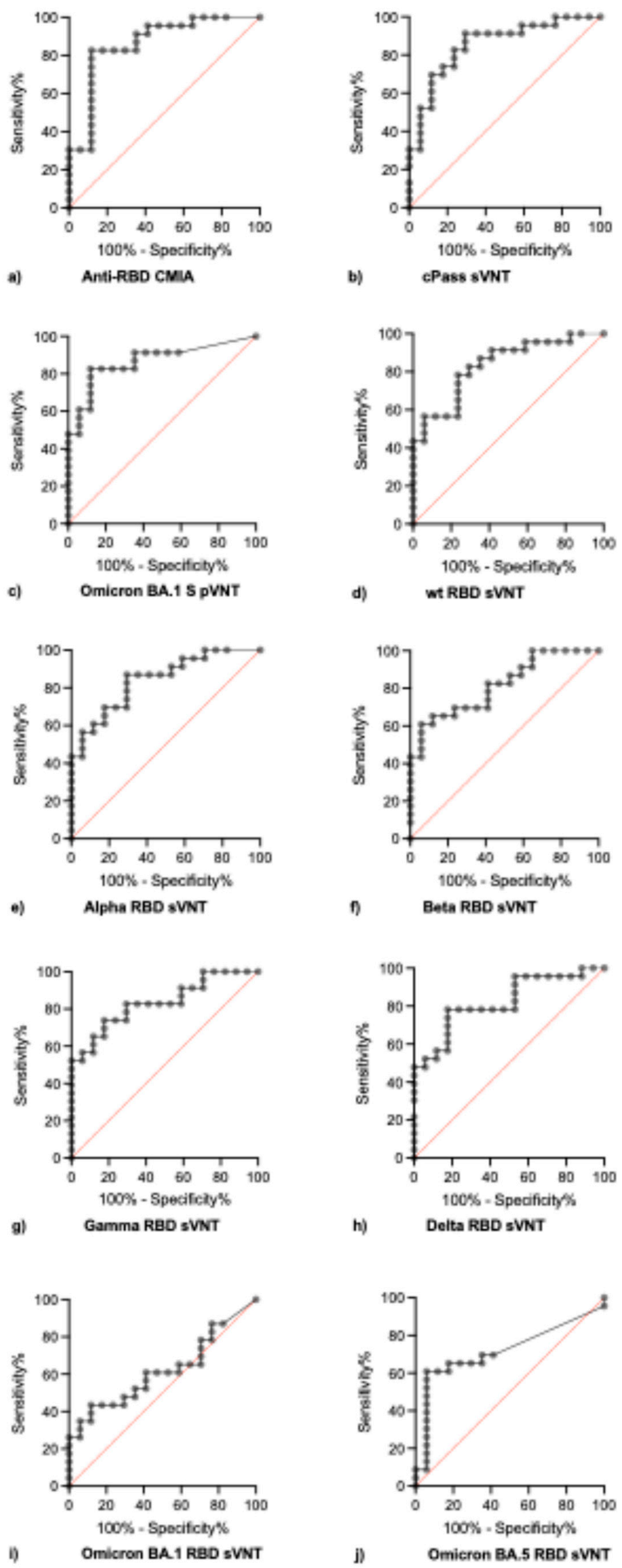

Figure S1. Analysis of sensitivity and specificity of assays (**a – j**) by ROC curve analysis using the results of the wt S pVNT as gold standard.
